# Open-chest versus closed-chest cardiopulmonary resuscitation in blunt trauma: analysis of a nationwide trauma registry

**DOI:** 10.1186/s13054-017-1759-1

**Published:** 2017-07-03

**Authors:** Akira Endo, Atsushi Shiraishi, Yasuhiro Otomo, Makoto Tomita, Hiroki Matsui, Kiyoshi Murata

**Affiliations:** 1 Trauma and Acute Critical Care Medical Center, Hospital of Medicine, Tokyo Medical and Dental University, 1-5-45 Yushima, Bunkyo-ku, Tokyo, 113-8510 Japan; 2 Emergency and Trauma Center, Kameda Medical Center, 929 Higashicho, Kamogawa, Chiba Japan; 3 Clinical Research Center, Hospital of Medicine, Tokyo Medical and Dental University, 1-5-45 Yushima, Bunkyo-ku, Tokyo, Japan; 4 Department of Clinical Epidemiology and Health Economics, School of Public Health, University of Tokyo, 7-3-1 Hongo, Bunkyo-ku, Tokyo, Japan

**Keywords:** Polytrauma, Resuscitation, Emergency thoracotomy, Cardiac arrest, Shock, Registry

## Abstract

**Background:**

Although open-chest cardiopulmonary resuscitation (OCCPR) is often considered as the last salvage maneuver in critically injured patients, evidence on the effectiveness of OCCPR has been based only on the descriptive studies of limited numbers of cases or expert opinions. This study aimed to compare the effectiveness of OCCPR with that of closed-chest cardiopulmonary resuscitation (CCCPR) in an emergency department (ED).

**Methods:**

A nationwide registry-based, retrospective cohort study was conducted. Patients with blunt trauma, undergoing cardiopulmonary resuscitation (CPR) in an ED between 2004 and 2015 were identified and divided into OCCPR and CCCPR groups. Their outcomes (survival to hospital discharge and survival over 24 hours following ED arrival) were compared with propensity score matching analysis and instrumental variable analysis.

**Results:**

A total of 6510 patients (OCCPR, 2192; CCCPR, 4318) were analyzed. The in-hospital and 24-hour survival rates in OCCPR patients were 1.8% (40/2192) and 5.6% (123/2192), and those in CCCPR patients were 3.6% (156/4318) and 9.6% (416/4318), respectively. In the propensity score-matched subjects, OCCPR patients (n = 1804) had significantly lower odds of survival to hospital discharge (odds ratio (95% CI)) = 0.41 (0.25–0.68)) and of survival over 24 hours following ED arrival (OR (95% CI) = 0.59 (0.45–0.79)) than CCCPR patients (n = 1804). Subgroup analysis revealed that OCCPR was associated with a poorer outcome compared to CCCPR in patients with severe pelvis and lower extremity injury.

**Conclusions:**

In this large cohort, OCCPR was associated with reduced in-hospital and 24-hour survival rates in patients with blunt trauma. Further comparisons between OCCPR and CCCPR using additional information, such as time course details in pre-hospital and ED settings, anatomical details regarding region of injury, and neurological outcomes, are necessary.

**Electronic supplementary material:**

The online version of this article (doi:10.1186/s13054-017-1759-1) contains supplementary material, which is available to authorized users.

## Background

Open-chest cardiopulmonary resuscitation (OCCPR) is often considered as one of the last salvage maneuvers in selected critically injured patients [1]; however, the procedure raises potential issues, including complications of the maneuver, cost-effectiveness, and exposure of medical staff to infection [2–5].

Recent guidelines recommended avoiding the indiscriminate use of emergency department (ED) thoracotomy (EDT) [6–8]. The indications for EDT in these guidelines are based upon a positive finding of signs of life (detectable blood pressure, respiratory or motor effort, cardiac electrical activity, or pupillary activity) or the time from onset of cardiac arrest to determine the utility of EDT. While guidelines have used the anatomical location of the injury as an indication for EDT in patients with penetrating trauma, no detailed description of the location of the injury is available for patients with blunt trauma. Although many reports have demonstrated an unfavorable outcome in patients with blunt trauma when undergoing OCCPR [9–11], it is important to note that there are several case reports suggesting that OCCPR may be effective in patients with blunt trauma [12, 13]. However, most of aforementioned evidence on EDT is based on descriptive studies of a limited number of patients, and the comparative effectiveness of OCCPR and closed-chest cardiopulmonary resuscitation (CCCPR) has remained unclear.

Recently, Suzuki et al. showed that patients undergoing thoracotomy within 24 hours of ED arrival had worse survival rates compared to patients undergoing CCCPR in an ED [14]. However, their results should be interpreted with caution because the study included patients undergoing thoracotomy for reasons other than for resuscitation. Furthermore, patients with cardiac arrest on ED arrival, normally requiring an evaluation of indications for OCCPR, were excluded from this study.

In developed countries, the majority of trauma patients are injured by blunt mechanisms; however, evidence on the clinical relevance of OCCPR for blunt trauma is limited compared to that on penetrating trauma [15]. The purpose of this study was to compare the effectiveness of OCCPR in the ED with that of CCCPR and to evaluate subgroups potentially benefitting from OCCPR.

## Methods

### Data source

The Japan Trauma Data Bank (JTDB) is a nationwide trauma registry established in 2003, and all trauma patients with an abbreviated injury scale (AIS) score ≥3 in any anatomical region are required to be registered. During the study period, the JTDB received records from 256 hospitals, of which 95% were government-approved tertiary emergency medical centers. The database includes information on injury mechanism, pre-hospital time course, patient baseline characteristics including vital signs at the scene of injury and on arriving at the ED, procedures performed, and status at hospital discharge (deceased or alive). Procedures performed in the ED are stipulated separately from other procedures performed in the operating theater after admission to the ward.

### Design and settings

This was a retrospective cohort study evaluating the effectiveness of OCCPR in patients with blunt trauma, using registry data available in the JTDB. We collected data on patients with blunt injury who underwent cardiopulmonary resuscitation (CPR) in the ED. The patients were divided into two groups according to whether or not OCCPR was performed in the ED. We compared the outcomes of the two groups, adjusting for clinical background using propensity score matching analysis. We also evaluated the potential benefits and drawbacks of OCCPR compared with CCCPR among subgroups. The ethics committee of Tokyo Medical and Dental University approved this study (#2192).

### Study population

We included patients with blunt trauma who received CPR in an ED from January 2004 to December 2015. We excluded patients with an AIS score of 6 because, by definition, these injuries are fatal (i.e. anatomically unsalvageable injury). Patients with a missing AIS score in any anatomical region were excluded to avoid analyzing the patients who potentially had an anatomically unsalvageable injury. Patients transferred from another hospital were also excluded.

### Data collection

We collected the following patient information from the JTDB: age, sex, year of injury, pre-hospital vital signs (systolic blood pressure, heart rate, and respiratory rate), time from emergency medical service (EMS) dispatch to ED arrival, pre-hospital treatment by paramedics (chest compression and defibrillation), vital signs (systolic blood pressure, heart rate, and respiratory rate) and Glasgow coma scale (GCS) score on ED arrival, AIS score for each region, injury severity score (ISS), resuscitation method (OCCPR or CCCPR), status at hospital discharge (survival or death), and time to death or hospital discharge. In addition, we collected hospital information, including the annual number of registered patients, OCCPR cases, and unexpected survivors defined as patients who had survived with a probability of survival based on the trauma and injury severity score (TRISS) <0.5 [16].

### Definitions and outcomes

Cardiac arrest was defined as a registered systolic blood pressure = 0 mmHg based on the JTDB data registration instructions, which say that systolic blood pressure in a patient whose pulse is not palpable should be registered as 0 mmHg even though actual blood pressure could not be 0 mmHg (i.e. cardiac arrest). The primary study outcome was survival to hospital discharge and the secondary study outcome was survival over 24 hours following arrival at the ED.

### Statistical analysis

Statistical analysis was performed using R 3.2.3 (R Foundation for Statistical Computing, Vienna, Austria). Missing mechanism of data in the naïve dataset were assumed clinically as missing at random (Additional file 1), so that missing data on the collected variables were complemented by the method of multivariate imputation by chained equations with 15 iterations using the package “mice” [17] and 51 datasets were produced. Descriptive statistics were used to display categorical variables as counts and percentages, and numeric or ordered variables as medians and 25^th^–75^th^ percentiles, after pooling all the imputed datasets into one dataset. Predictive statistics were used to display the estimators as point estimation and 95% confidence intervals (CI) integrated across the imputed datasets, based on Rubin’s rule [18].

We used propensity score matching to compare the outcomes between the OCCPR group and the CCCPR group. The propensity score for predicting OCCPR was calculated by logistic regression analysis using variables pertaining to the year of injury, patient factors (age and sex), time from EMS dispatch to ED arrival, mean number of unexpected survivors per year in the treating hospital that the patient was transferred to, vital signs at the scene of injury (systolic blood pressure, heart rate, and respiratory rate), whether or not cardiac arrest at the scene of injury was observed, vital signs (systolic blood pressure, heart rate, respiratory rate, and body temperature) and GCS on ED arrival, whether or not cardiac arrest was observed on ED arrival, AIS score of each region, and ISS. Propensity score matching extracted 1:1 matched pairs of subjects from the OCCPR group and the CCCPR group using the values of the logit-transformed propensity score calculated for each imputed dataset and averaged across the datasets. Match balance between the two groups was assessed by the absolute standardized mean difference of all the variables, and values lower than 0.1 were regarded as acceptable. To achieve this match balance, the caliper width of matching was set at 0.01. Intergroup comparison of the outcomes with propensity-score-matched subjects was performed using the chi-square test. Survival curves were constructed using Kaplan-Meier estimates for the propensity-score-matched subjects and were compared using a log-rank test.

The primary outcome was compared in the propensity-score-matched cohort across subgroups stratified according to sex, age (<50 or ≥50 years), time from EMS dispatch to ED arrival (<30 or ≥30 min), systolic blood pressure on ED arrival (<60 or ≥60 mmHg), whether or not cardiac arrest was observed at the scene of injury, whether or not cardiac arrest was observed on ED arrival, ISS (<30 or ≥30), and AIS score of head, chest, abdomen, and pelvis and lower extremities (0–2 or 3–5). For each subgroup, logistic regression analysis was used to estimate the odds ratio (OR) and interaction for survival to hospital discharge in the OCCPR group and the CCCPR group.

We performed instrumental variable analysis, which is an established technique used to control unmeasured confounding in nonrandomized data [19], as the sensitivity analysis for the propensity score matching. This approach was conducted using a two-stage least-squares regression analysis adjusted by following variables: year of injury, sex, TRISS, systolic blood pressure at the scene of injury, and AIS of head, chest, abdomen, and pelvis and lower extremities, on multiply imputed and not propensity score-matched dataset. The mean number of registered patients per year in the hospital was also incorporated into the model as a measure of quality of trauma care provided by the hospital [20]. Issues with variable multicollinearity were assessed by variance inflation factor (VIF) analysis and the tolerance value was set at <2. We selected the variable of “the mean number of OCCPR cases per year in the hospital” as the instrumental variable. The null hypothesis was that there was no association between the mean number of OCCPR cases per year in the hospital and the actual implementation of OCCPR. A partial *F* test was conducted to assess an issue of weak instruments, and a value of *F*-statistic >10 was regarded as acceptable. The level of significance was defined as *p* < 0.05 for all statistical analyses.

## Results

### Study population

**Fig. 1:**
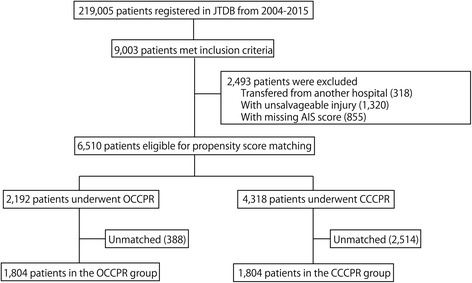
Patient selection. *JTDB* Japan Trauma Databank, *AIS* abbreviated injury scale, *OCCPR* open-chest cardiopulmonary resuscitation, *CCCPR* closed-chest cardiopulmonary resuscitation

The flow diagram of the patient selection process is shown in Fig. 1. A total of 6510 patients who received CPR in the ED were identified and divided into the OCCPR group (n = 2192) and the CCCPR group (n = 4318), from which 1804 propensity-score-matched pairs were generated.

### Patient characteristics

The baseline characteristics of the multiply imputed dataset before and after propensity score matching is shown in Table 1. The distribution of naïve data and the proportions of missing values are shown in Additional file 2.

**Table 1:** Baseline characteristics of the multiply imputed dataset before and after propensity score matching

	Unmatched cohort			Matched cohort		
Variables	Open-chest CPR	Closed-chest CPR	SMD	Open-chest CPR	Closed-chest CPR	SMD
Number of subjects	2192	4318		1804	1825	
Age (years)	52 (34–68)	56 (36–73)	0.12	52 (34–68)	53 (34–71)	0.05
Sex, male, *n* (%)	1533 (69.9)	2865 (66.4)	0.08	1250 (69.3)	1248 (69.2)	0.00
Year of Injury, *n* (%)						
2004	65 (3.0)	170 (3.9)	0.05	63 (3.5)	63 (3.5)	0.00
2005	58 (2.6)	181 (4.2)	0.08	56 (3.1)	57 (3.1)	0.00
2006	77 (3.5)	168 (3.9)	0.02	75 (4.2)	56 (3.1)	0.06
2007	106 (4.8)	323 (7.5)	0.11	100 (5.5)	94 (5.2)	0.01
2008	155 (7.1)	319 (7.4)	0.01	144 (8.0)	126 (7.0)	0.04
2009	155 (7.1)	313 (7.2)	0.01	144 (8.0)	135 (7.5)	0.02
2010	273 (12.5)	468 (10.8)	0.05	249 (13.8)	232 (12.9)	0.03
2011	240 (10.9)	541 (12.5)	0.05	216 (13.0)	203 (11.3)	0.02
2012	300 (13.7)	544 (12.6)	0.03	251 (13.9)	251 (13.9)	0.00
2013	283 (12.9)	542 (12.6)	0.01	223 (12.4)	226 (12.5)	0.01
2014	267 (12.2)	423 (9.8)	0.08	161 (8.9)	201 (11.1)	0.07
2015	213 (9.7)	326 (7.5)	0.08	122 (6.8)	160 (8.9)	0.08
Vital signs at the scene of injury						
Systolic blood pressure, mmHg	76 (0–109)	67 (0–107)	0.11	71 (0–107)	68 (0–104)	0.06
Heart rate, beats/min	60 (0–108)	0 (0–90)	0.28	53 (0–100)	30 (0–100)	0.06
Respiratory rate, breaths/min	10 (0–24)	0 (0–20)	0.26	6 (0–24)	0 (0–24)	0.05
Cardiac arrest at the scene of injury, *n* (%)	798 (36.4)	1881 (43.6)	0.15	714 (39.6)	771 (42.7)	0.06
Pre-hospital treatment, *n* (%)						
Chest compression	1045 (47.7)	2518 (58.3)	0.21	935 (51.8)	943 (52.3)	0.01
Defibrillation	34 (1.6)	106 (2.5)	0.06	31 (1.7)	36 (2.0)	0.02
Time from EMS dispatch to ED arrival, mins	33 (25–45)	32 (25–42)	0.00	32 (25–44)	32 (25–44)	0.01
Vital signs on ED arrival						
Systolic blood pressure, mmHg	0 (0–40)	0 (0–40)	0.00	0 (0–40)	0 (0–40)	0.01
Heart rate, beats/min	0 (0–84)	0 (0–60)	0.16	0 (0–75)	0 (0–72)	0.01
Respiratory rate, breaths/min	0 (0–15)	0 (0–0)	0.14	0 (0–8)	0 (0–10)	0.05
Body temperature, °C	35.2 (34.2–36.0)	35.2 (34.2–36.1)	0.00	35.2 (34.2–36.0)	35.2 (34.2–36.0)	0.02
Glasgow coma scale on ED arrival	3 (3–3)	3 (3–3)	0.07	3 (3–3)	3 (3–3)	0.02
Revised trauma score on ED arrival	0.00 (0.00–2.34)	0.00 (0.00–1.47)	0.06	0.00 (0.00–1.90)	0.00 (0.00–1.90)	0.00
Cardiac arrest on ED arrival, *n* (%)	1480 (67.5)	3110 (72.0)	0.10	1266 (70.2)	1272 (70.5)	0.01
Abbreviated injury scale						
Head	0 (0–3)	3 (0–4)	0.47	0 (0–3)	0 (0–3)	0.04
Face	0 (0–0)	0 (0–0)	0.12	0 (0–0)	0 (0–0)	0.01
Neck	0 (0–0)	0 (0–0)	0.02	0 (0–0)	0 (0–0)	0.01
Chest	4 (3–5)	4 (0–5)	0.37	4 (3–5)	4 (3–5)	0.01
Abdomen	0 (0–2)	0 (0–0)	0.37	0 (0–0)	0 (0–0)	0.02
Spine	0 (0–0)	0 (0–0)	0.07	0 (0–0)	0 (0–0)	0.00
Upper extremities	0 (0–0)	0 (0–1)	0.03	0 (0–1)	0 (0–1)	0.01
Pelvis and lower extremities	2 (0–3)	2 (0–3)	0.14	2 (0–3)	2 (0–3)	0.02
Surface	0 (0–0)	0 (0–0)	0.09	0 (0–0)	0 (0–0)	0.00
Injury severity score	34 (25–43)	29 (22–41)	0.22	34 (25–43)	34 (25–43)	0.02
Probability of survival, %	3.6 (1.1–14.4)	3.6 (1.2–13.4)	0.01	3.6 (1.2–13.4)	3.6 (1.0–13.4)	0.00

Numeric variables are expressed as median (25^th^–75^th^ percentiles). *Abbreviations*: *CPR* cardiopulmonary resuscitation, *SMD* standardized mean difference, *EMS* emergency medical services, *ED* emergency department

The in-hospital and 24-hour survival rates in the OCCPR group were 1.8% (40/2192) and 5.6% (123/2192), and those in the CCCPR group were 3.6% (156/4318) and 9.6% (416/4318), respectively.

### Propensity score matching

The standardized mean difference in the variables according to the estimated propensity score indicated a well-matched balance (Table 1). Of the propensity-score-matched subjects, the proportion of patients surviving to hospital discharge in the OCCPR and CCCPR groups were 1.2% (22/1804) and 3.3% (60/1804), respectively. The proportion of patients in each group surviving over 24 hours after ED arrival were 4.9% (89/1804) and 8.1% (147/1804), respectively. The OCCPR group had significantly lower odds of survival to hospital discharge than the CCCPR group (OR (95% CI) = 0.41 (0.25–0.68)), and the OCCPR group had significantly lower odds of survival over 24 hours after ED arrival than the CCCPR group (OR (95% CI) = 0.59 (0.45–0.79); Table 2).

**Table 2:** Results of analysis of study outcomes using propensity score matching

Outcomes	Number of patients (%) OCCPR/CCCPR	Odds ratio (95% confidence interval)	*P* value
Unmatched cohort (OCCPR, 2192 patients; CCCPR, 4318 patients)			
Survival to hospital discharge	40 (1.8)/156 (3.6)	0.49 (0.34–0.71)	<0.001
Survival over 24 hours after ED arrival	123 (5.6)/416 (9.6)	0.56 (0.45–0.70)	<0.001
Matched cohort (OCCPR, 1804 patients; CCCPR, 1804 patients)			
Survival to hospital discharge	22 (1.2)/60 (3.3)	0.41 (0.25–0.68)	<0.001
Survival over 24 hours after ED arrival	89 (4.9)/147 (8.1)	0.59 (0.45–0.79)	<0.001

*Abbreviations*: *OCCPR* open-chest cardiopulmonary resuscitation, *CCCPR* closed-chest cardiopulmonary resuscitation, *ED* emergency department

**Fig. 2:**
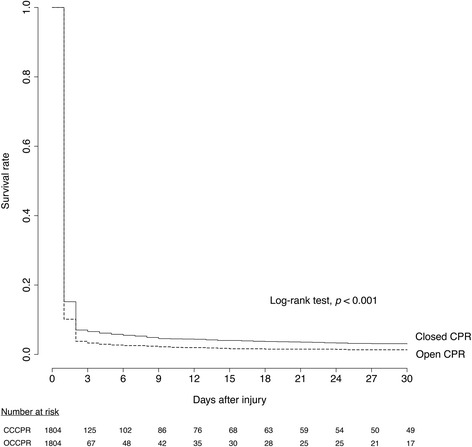
Kaplan-Meier analysis of 30-day mortality in propensity-score-matched subjects. *OCCPR* open-chest cardiopulmonary resuscitation, *CCCPR* closed-chest cardiopulmonary resuscitation

Survival curve analysis of 30-day mortality was performed (Fig. 2). The log-rank test results revealed significant superiority of CCCPR compared to OCCPR (*p* < 0.001).

### Subgroup analysis

**Fig. 3:**
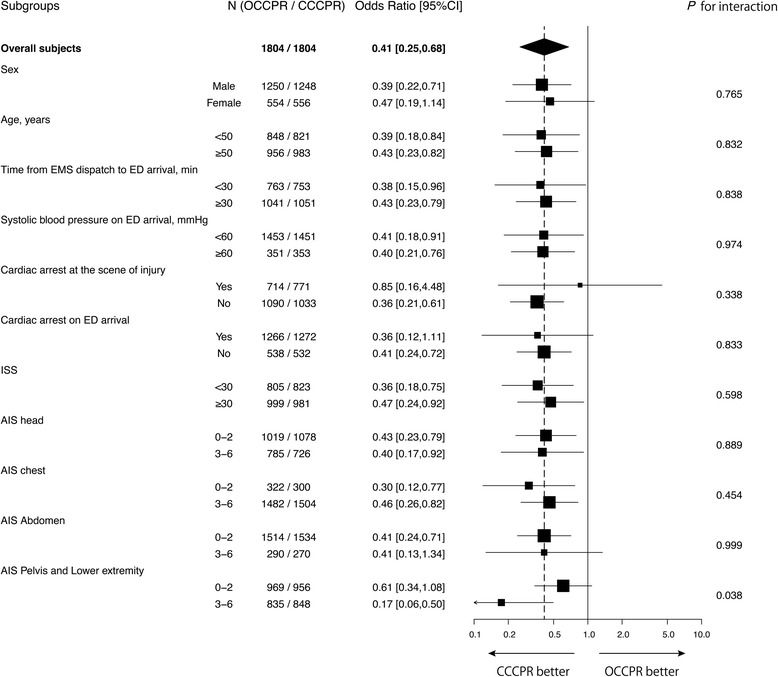
The results of subgroup analysis for the primary outcome. Odds ratios for survival to hospital discharge [95% confidence interval] in each subgroup and *p* values for interaction between subgroups are presented. *OCCPR* open-chest cardiopulmonary resuscitation, *CCCPR* closed-chest cardiopulmonary resuscitation, *CI* confidence interval, *EMS* emergency medical services, *ED* emergency department, *ISS* injury severity score, *AIS* abbreviated injury scale

Results of the subgroup analysis are shown in Fig. 3. There were no statistically significant differences between dichotomized subgroups in any variables except for AIS score for pelvic and lower extremity injuries. In patients with severe pelvic and lower extremity injury (AIS score ≥3), OCCPR was significantly associated with a poor outcome compared to CCCPR (*p* for interaction = 0.038).

### Sensitivity analysis

All of the VIFs of variables used in the linear regression analysis were lower than 2, which eliminated the issue of multicollinearity in our model. The linear regression analysis demonstrated that in one case of an increase in the instrumental variable, there was a significant increase in the proportion of OCCPR implementation (mean difference (95% CI) = 2.9% (2.7, 3.1), *F* statistic = 74.1); therefore, the null hypothesis that there was no association between the mean number of OCCPR cases per year in the hospital and actual implementation of OCCPR was rejected. However, survival to hospital discharge and survival over 24 hours after ED arrival were not significantly affected by the instrumental variable in the linear regression analysis adjusted by OCCPR (mean difference (95% CI) = 0.0% (−0.0, 0.0) and mean difference (95% CI) = 0.1% (−0.0, 0.2), respectively). Therefore, the variable “the mean number of OCCPR cases per year in the hospital” satisfied the requirements of the instrumental variable in this sensitivity analysis.

The two-way least-squares analysis with this instrumental variable did not alter the original analysis by propensity score matching (mean difference (95% CI) = –5.0% (−9.2, –0.8) for survival to hospital discharge and mean difference (95% CI) = –5.6% (−12.3, 1.1) for survival over 24 hours after ED arrival, respectively; Table 3).

**Table 3:** Results of instrumental variable analysis of study outcomes

Outcomes	Number of patients (%) OCCPR/CCCPR	Adjusted difference (%) (95% confidence interval)	*P* value
Survival to hospital discharge	40 (1.8)/156 (3.6)	–5.0 (–9.2, –0.8)	0.019
Survival over 24 hours after ED arrival	123 (5.6)/416 (9.6)	–5.6 (−12.3, 1.1)	0.100

*Abbreviations*: *OCCPR* open-chest cardiopulmonary resuscitation, *CCCPR* closed-chest cardiopulmonary resuscitation, *ED* emergency department

## Discussion

In this nationwide trauma registry, we evaluated the comparative effectiveness of OCCPR in the ED with that of CCCPR in patients with blunt trauma, adjusting for the available patient characteristics. OCCPR was associated with worse rates of survival to hospital discharge and of survival over 24 hours after ED arrival. To our knowledge, there have been no reports comparing OCCPR and CCCPR in patients with cardiac arrest on ED arrival. A prospective randomized controlled trial assessing the comparative effectiveness of OCCPR is not feasible due to ethical concerns; therefore, this analysis of a large database is of significant value in the context of trauma resuscitation.

Although we made strong efforts to control possible biases by incorporating as many available variables as possible into the model and using several statistical techniques, the possibility remained that additional factors not identified in this study potentially influenced clinicians to choose OCCPR instead of CCCPR. If the assumption was valid, patients in the OCCPR group and patients in the CCCPR group would be entirely different cohorts and could not be compared. To evaluate the feasibility of comparing the OCCPR and CCCPR groups, we assessed the proportion of OCCPR implementation among the total CPR cases in each treating hospital. The results showed that the proportion of OCCPR implementation varied greatly depending on each treating hospital (Additional file 3), suggesting that the indications for OCCPR were likely hospital-dependent. It was therefore possible that some patients met the indications for both OCCPR and CCCPR, but were relegated to one group or the other based on the standard operating procedures of the treating hospital. Considering this patient and hospital background, the design of this study that compared the effectiveness of resuscitative approaches (i.e. OCCPR or CCCPR) in patients who met the indications for both OCCPR and CCCPR was reasonable.

Survival rates among patients with blunt trauma undergoing EDT have been reported as 1.4–1.6% [7, 21]. The 1.8% survival rate among patients undergoing OCCPR in the present study was comparable. Therefore, the population in this study may not be biased in terms of survival rates. The reason for the worse survival rates in patients receiving OCCPR in this study may be partially explained by the fact that OCCPR is more invasive than CCCPR, resulting in an additional insult to a critically injured patient. Another major reason may be the loss of the forward blood flow function caused by the loss of intrathoracic pressure. Direct heart compression without sufficient intrathoracic pressure (i.e. thoracic pump function) may have harmful circulatory effects. Furthermore, the JTDB does not provide information on the duration of CCCPR. Therefore, considering the situation that OCCPR was performed as the second-line therapy following CCCPR, the patients who achieved return of spontaneous circulation over a short period of time with CCCPR only were assigned to the CCCPR group in this study, and it is possible that those patients improved the survival rates of the CCCPR group. The lack of this information is an important limitation to our study.

In the subgroup analysis, despite the theoretical benefit of temporary hemostasis by aortic cross-clamping, OCCPR was associated with decreased survival rates in patients with severe injuries of the pelvis and lower extremities. Furthermore, we could not demonstrate the superiority of OCCPR in patients with severe abdominal injury. In cases of abdominal or pelvic injury, it is possible that temporary hemostasis of distal organs was achieved more effectively by alternative approaches such as resuscitative endovascular balloon occlusion of the aorta or cross-clamping of the abdominal aorta during laparotomy in the CCCPR group. Further characterization of these differences would be useful for identifying patients who would potentially benefit from OCCPR.

In this study, we could not conclude that OCCPR was superior to CCCPR in patients without cardiac arrest on ED arrival, despite this subset of patients being previously described as ideal candidates for OCCPR [21, 22]. This result was consistent with the findings of Suzuki et al. [14]. Occasionally OCCPR is performed as a last resort in patients with catastrophic hemorrhage despite exhaustion of all other hemostatic maneuvers. In most of these cases it is unlikely that OCCPR could result in a favorable outcome. The superiority of OCCPR compared to CCCPR in patients without cardiac arrest on ED arrival may be lower than previously considered.

There are also reports of cases in which patient survival was dependent on OCCPR [23, 24], suggesting the existence of a specific population that may benefit from OCCPR. Although this study analyzed a larger number of patients than in previous studies, there were only 40 survivors after OCCPR. We could not identify the specific subgroups that benefit from OCCPR because of the limited number of survivors, and further studies including more survivors are required.

Our study has several limitations. The study design was retrospective; however, our research question cannot feasibly be assessed in a randomized controlled trial because of the ethical issue. Further, because of the nature of the JTDB, important information, such as data on the duration of CCCPR and data on cardiac rhythm at thoracotomy, which were considered important predictors in other reports [25, 26], were unavailable. The data on neurological outcome, which is the ultimate outcome of CPR, was also unavailable in the JTDB. In addition, there was some degree of missing data in the JTDB, particularly on pre-hospital vital signs (Additional file 2). Although our methodology in handling missing data was considered reasonable and statistically appropriate, the results should be interpreted with caution owing to the missing data. Despite these limitations, our well-designed retrospective study provided notable insight into the field of trauma resuscitation, as the comparative effectiveness of OCCPR and CCCPR have never been reported.

## Conclusions

OCCPR was associated with reduced rates of survival to hospital discharge and of survival over 24 hours after ED arrival in patients with blunt trauma; and the study could not identify a specific subpopulation that would benefit from OCCPR. Further comparisons between OCCPR and CCCPR with larger numbers of patients, using additional information, such as time course details in pre-hospital and in ED settings, anatomical details on the region of injury, and neurological outcomes, are necessary.

## Additional files

Additional file 1: Details of missing data handling. The evidence of the assumption od data being missing at random in the naïve dataset is described. (DOCX 59 kb)

Additional file 2: Baseline characteristics and proportion of missing data in the naïve dataset. (DOCX 24 kb)

Additional file 3: Number of open-chest cardiopulmonary resuscitations and/or closed-chest cardiopulmonary resuscitations at hospitals stratified by a proportion of open-chest cardiopulmonary resuscitation cases among the total cardiopulmonary resuscitation cases. *OCCPR* open-chest cardiopulmonary resuscitation, *CCCPR* closed-chest cardiopulmonary resuscitation. (TIF 8691 kb)

## Abbreviations

AIS: Abbreviated injury scale
CCCPR: Closed-chest cardiopulmonary resuscitation
CI: Confidence interval
CPR: Cardiopulmonary resuscitation
ED: Emergency department
EDT: Emergency department thoracotomy
EMS: Emergency medical system
GCS: Glasgow coma scale
ISS: Injury severity score
JTDB: Japan Trauma Data Bank
OCCPR: Open-chest cardiopulmonary resuscitation
OR: Odds ratio
TRISS: Trauma and injury severity score
VIF: Variance inflation factor
