# Supplementary material for: Open-chest versus closed-chest cardiopulmonary resuscitation in blunt trauma: analysis of a nationwide trauma registry
Source: Crit Care. 2017 Jul 3;21:169. doi: 10.1186/s13054-017-1759-1 (PMC5496413; doi:10.1186/s13054-017-1759-1)

**Additional file 1**

***Missing data handling***

The collected data in this study contained missing values especially in the vital signs at the scene of injury. There is no established method to identify the missing mechanism statistically, however, occurred missingness in this study was considered to be strongly related to the other observed variables from clinical perspective. For example, systolic blood pressure at the scene of injury was considered to be related to heart rate and respiratory rate at the scene of injury or pre-hospital treatment such as chest compression. Further, considering the possibility that the missing data might be affected by the missing value itself (i.e. Missing not at random), we collected various variables expected to be related to the missing values and incorporated them into the imputation model to approximate missing mechanism to MAR according to inclusive analysis strategy (1, 2).

The figures for distribution of the observed and the imputed value in this study for systolic blood pressure at the scene of injury were shown in below. These histograms indicated that the distribution of imputed values was obviously different compared to that of observed values. These data indicated that the missingness in this study were significantly affected by the other observed variables, so that the missing could be assumed as MAR, which is the good indication for multiple imputation method.

(1) Enders CK (2002) Applied Missing Data Analysis. New York: Guilford Press

(2) Schafer JL, Graham JW (2002) Missing data: our view of the state of the art. Psychol Methods 7:147-177


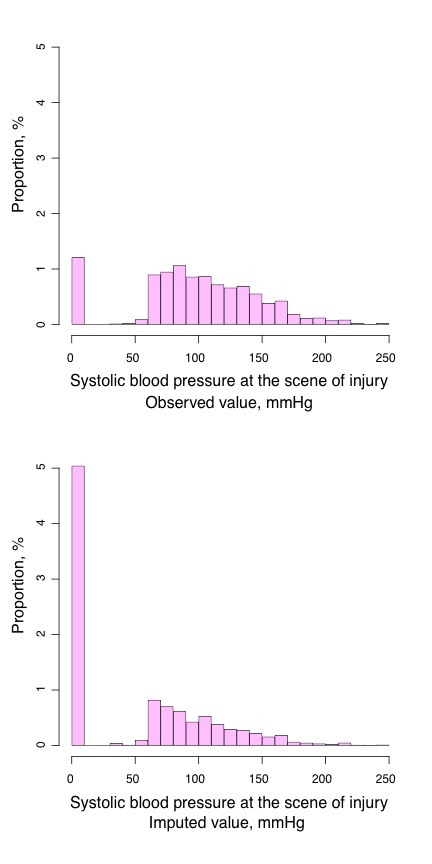

Supplement: Supplementary file 1 — Details of missing data handling. The evidence of the assumption od data being missing at random in the naïve dataset is described. (DOCX 59 kb) [file 13054_2017_1759_MOESM1_ESM.docx]
