# Supplementary material for: Open-chest versus closed-chest cardiopulmonary resuscitation in blunt trauma: analysis of a nationwide trauma registry
Source: Crit Care. 2017 Jul 3;21:169. doi: 10.1186/s13054-017-1759-1 (PMC5496413; doi:10.1186/s13054-017-1759-1)
Supplement: Supplementary file 2 — Baseline characteristics and proportion of missing data in the naïve dataset. (DOCX 24 kb) [file 13054_2017_1759_MOESM2_ESM.docx]

| **Additional file 2. Baseline characteristics and proportion of missing data in naïve dataset** | | | | | |
| --- | --- | --- | --- | --- | --- |
| Variables | | Open-chest (n= 2192) | | Close-chest (n= 4318) | |
|  |  | Registered data | Missing, n (%) | Registered data | Missing, n (%) |
| Age (years) | | 52 [34–68] | 12 (0.5) | 56 [36–73] | 24 (0.6) |
| Sex, Male, n (%) | | 1533 (70.0) | 0 (0) | 2865 () | 0 (0) |
| Year of injury, n (%) | |  | 4 (0.2) |  | 17 (0.4) |
|  | 2004 | 65 (3.0) |  | 170 (3.9) |  |
|  | 2005 | 58 (2.6) |  | 180 (4.2) |  |
|  | 2006 | 77 (3.5) |  | 167 (3.9) |  |
|  | 2007 | 106 (4.8) |  | 322 (7.5) |  |
|  | 2008 | 155 (7.1) |  | 318 (7.4) |  |
|  | 2009 | 155 (7.1) |  | 312 (7.2) |  |
|  | 2010 | 273 (12.5) |  | 466 (10.8) |  |
|  | 2011 | 240 (10.9) |  | 539 (12.5) |  |
|  | 2012 | 299 (13.6) |  | 542 (12.6) |  |
|  | 2013 | 282 (12.9) |  | 540 (12.5) |  |
|  | 2014 | 266 (12.1) |  | 421 (9.7) |  |
|  | 2015 | 212 (9.7) |  | 324 (7.5) |  |
| Vital signs at the scene of injury | |  |  |  |  |
|  | Systolic blood pressure, mmHg | 99 [78–129] | 1621 (74.0) | 101 [73–138] | 3317 (76.8) |
|  | Heart rate, beats/min | 80 [0–120] | 900 (41.1) | 57 [0–100] | 2050 (47.5) |
|  | Respiratory rate, breaths/min | 12 [0–30] | 877 (40.0) | 4 [0–24] | 2075 (48.1) |
| Cardiac arrest at the scene of injury | | 45 (2.1) | 1621 (74.0) | 145 (14.7) | 3317 (76.8) |
| Pre-hospital treatment | |  |  |  |  |
|  | Chest compression　(%) | 967 (44.1) | 209 (9.5) | 2375 () | 303 (7.0) |
|  | Defibrillation (%) | 30 (1.4) | 209 (9.5) | 95 () | 303 (7.0) |
| Time from EMS dispatch to ED arrival, min | | 33 [25–46] | 748 (34.1) | 31 [24–42] | 1394 (32.3) |
| Vital signs on ED arrival | |  |  |  |  |
|  | Systolic blood pressure, mmHg | 0 [0–40] | 98 (4.5) | 0 [0–40] | 179 (4.1) |
|  | Heart rate, beats per min | 0 [0–85] | 180 (8.2) | 0 [0–64] | 359 (8.3) |
|  | Respiratory rate, breaths/min | 0 [0–12] | 207 (9.4) | 0 [0–0] | 501 (11.6) |
|  | Body temperature, °C | 35.3 [34.4–36.0] | 1204 (54.9) | 35.3 [34.2–36.1] | 2183 (50.6) |
| Glasgow coma scale on ED arrival | | 3 [3–3] | 105 (4.8) | 3 [3–3] | 278 (6.4) |
| Revised trauma score on ED arrival | | 0.00 [0.00, 1.90] | 292 (13.3) | 0.00 [0.00–0.73] | 664 (15.4) |
| Cardiac arrest on ED arrival | | 1417 (64.6) | 98 (4.5) | 2986 (69.2) | 179 (4.1) |
| Abbreviated injury scale | |  | 0 (0) |  | 0 (0) |
|  | Head | 0 [0–3] |  | 3 [0–4] |  |
|  | Face | 0 [0–0] |  | 0 [0–0] |  |
|  | Neck | 0 [0–0] |  | 0 [0–0] |  |
|  | Chest | 4 [3–5] |  | 4 [0–5] |  |
|  | Abdomen | 0 [0–2] |  | 0 [0–0] |  |
|  | Spine | 0 [0–0] |  | 0 [0–0] |  |
|  | Upper extremities | 0 [0–0] |  | 0 [0–1] |  |
|  | Pelvis and lower extremities | 2 [0–3] |  | 2 [0–3] |  |
|  | Surface | 0 [0–0] |  | 0 [0–0] |  |
| Injury Severity Score | | 34 [25–43] | 0 (0) | 29 [22–41] | 0 (0) |
| Probability of Survival, % | | 3.6 [1.0–13.5] | 302 (13.8) | 3.6 [1.1–12.4] | 685 (15.9) |
| Numeric variables are expressed as median [25^th^–75^th^ percentiles].  Abbreviations: EMS, emergency medical system; ED, emergency department. | | | | | |
